# Spectral characteristics of the mutant form GGBP/H152C of D-glucose/D-galactose-binding protein labeled with fluorescent dye BADAN: influence of external factors

**DOI:** 10.7717/peerj.275

**Published:** 2014-03-18

**Authors:** Alexander V. Fonin, Olga V. Stepanenko, Olga I. Povarova, Catherine A. Volova, Elizaveta M. Philippova, Grigory S. Bublikov, Irina M. Kuznetsova, Alexander P. Demchenko, Konstantin K. Turoverov

**Affiliations:** 1 Laboratory of Structural Dynamics, Stability and Folding of Proteins, Institute of Cytology, Russian Academy of Science, St. Petersburg, Russia; 2 Department of Biology, St. Petersburg State University, St. Petersburg, Russia; 3 Department of Physical Electronics, St. Petersburg State Polytechnical University, St. Petersburg, Russia; 4 Department of Biophysics, St. Petersburg State Polytechnical University, St. Petersburg, Russia; 5 Laboratory of Nanobiotechnologies, Palladin Institute of Biochemistry of the National Academy of Sciences of Ukraine, Kiev, Ukraine

**Keywords:** D-glucose/D-galactose-binding protein, Fluorescent dye BADAN, Protein stability, Biosensors, Glucose binding constant

## Abstract

The mutant form GGBP/H152C of the D-glucose/D-galactose-binding protein with the solvatochromic dye BADAN linked to cysteine residue Cys 152 can be used as a potential base for a sensitive element of glucose biosensor system. We investigated the influence of various external factors on the physical-chemical properties of GGBP/H152C-BADAN and its complex with glucose. The high affinity (*K_d_* = 8.5 µM) and high binding rate of glucose make GGBP/H152C-BADAN a good candidate to determine the sugar content in biological fluids extracted using transdermal techniques. It was shown that changes in the ionic strength and pH of solution within the physiological range did not have a significant influence on the fluorescent characteristics of GGBP/H152C-BADAN. The mutant form GGBP/H152C has relatively low resistance to denaturation action of GdnHCl and urea. This result emphasizes the need to find more stable proteins for the creation of a sensitive element for a glucose biosensor system.

## Introduction

Operation of most of the glucose biosensors for continuous, non-invasive glucose monitoring in biological fluids is based on specific enzymatic glucose oxidation by glucose oxidase (Tang et al., 2004) or phosphorylation by glucokinase (Katsube et al., 1990). The main drawback of such sensors is that its sensitive element is consumable due to the irreversible nature of the reaction of sensitive elements with analyte. A more promising direction for non-invasive glucometer design is the development of a biosensor system with a protein-sensitive element those reaction with glucose is reversible. Such proteins are concanavalin A and the D-glucose/D-galactose-binding protein (GGBP). The application of GGBP is promising because the protein–glucose binding promotes a significant change in the tertiary structure of GGBP (Amiss et al., 2007; Sakaguchi-Mikami et al., 2008; Saxl et al., 2009; Yesylevskyy, Kharkyanen & Demchenko, 2006). One of the most common methods for detecting conformational changes in proteins is fluorescence. Despite the significant structural changes of GGBP that occur when it interacts with glucose the intrinsic UV fluorescence characteristics remains practically unchanged (Stepanenko et al., 2011a; Stepanenko et al., 2011b). Thus, the fluorescent response to glucose binding to GGBP could be obtained using fluorescence of dye linked to protein (Amiss et al., 2007; Khan, Gnudi & Pickup, 2008; Khan, Saxl & Pickup, 2010; Sakaguchi-Mikami et al., 2008; Saxl et al., 2011; Saxl et al., 2009) or the changes of FRET signal of chimeric constructs based on GGBP and fluorescent proteins (Ge, Tolosa & Rao, 2004; Ye & Schultz, 2003).

One of the promising candidates for the role of a glucose biosensor system sensitive element is the GGBP/H152C mutant form of GGBP in which the histidine at position 152 has been replaced with a cysteine and fluorescent dye BADAN linked to the Cys 152 (Khan, Gnudi & Pickup, 2008). It was shown that the fluorescence intensity of the dye linked to the protein increased three-fold in response to binding of GGBP/H152C with glucose (the transition of GGBP/H152C from its open to closed forms) (Khan, Gnudi & Pickup, 2008; Khan, Saxl & Pickup, 2010). The replacement of histidine 152 with cysteine was used for labeling with the fluorescent dye of series of GGBP mutant forms for glucose monitoring (Khan, Saxl & Pickup, 2010; Saxl et al., 2011). Continuous glucose concentration measurements in analyzable mediums could be achieved using a protein-sensitive element having as much as possible, a high stability, and which properties do not significantly change on the possible composition variations of the biological fluids, in which to determine glucose.

In this connection aim of this work was to study the effect of different external factors (i.e., changing the pH, viscosity, ionic strength of solution) on the fluorescence characteristics of the GGBP/H152C mutant in its open and closed forms and on the response time of the recorded signal to glucose change in the assay medium. One way to assess the stability of proteins is to study their unfolding under the action of chemical denaturants. Thus we also investigated denaturation GGBP/H152C under the GdnHCl and urea action.

## Materials and Methods

### Materials

D-glucose, acetonitrile (Sigma, USA), NaCl (Vekton, Russia), fluorescent dyes BADAN and quinine sulfate (AnaSpec, USA), guanidine hydrochloride (Nacalai Tesque, Japan), and glycerol (Merck, Germany) were used without further purification. The mutant form of the D-glucose/D-galactose–binding protein GGBP/H152C was obtained, separated and purified as described previously (Stepanenko et al., 2011a). The labeling of GGBP/H152C with the fluorescent dye BADAN was performed as described by Khan, Saxl & Pickup (2010). The experiments were performed in solutions with protein concentrations ranging from 0.2 to 0.8 mg/ml. For the formation of the protein-ligand complex, 5 µM–20 mM of D-glucose was added to the protein solution. The measurements were made in buffer solutions containing citric acid and Na_2_HPO_4_ (pH 2.8, 4.2, 6.0, 7.1), PBS (pH 7.4) and TrisHCl (pH 7.2, 9.6).

### Fluorescence measurements

The fluorescence experiments were carried out using Cary Eclipse (Agilent, Australia) and homemade (Turoverov et al., 1998) spectrofluorimeters. The kinetics of GGBP/H152C-BADAN binding with glucose was measured using a stopped flow apparatus MOS 450 (Bio-Logic, France). The excitation wavelengths for the intrinsic protein fluorescence spectra were 297 or 280 nm. The dye fluorescence was excited at 387 or 405 nm. The position and form of the fluorescence spectra were characterized by the parameter *A* = *I*_320_/*I*_365_, where *I*_320_ and *I*_365_ are the fluorescence intensities measured at emission wavelengths of 320 and 365 nm, respectively (Kuznetsova, Yakusheva & Turoverov, 1999; Turoverov & Kuznetsova, 2003). The values of parameter *A* and the fluorescence spectra were corrected using the instrument’s spectral sensitivity. The quantum yield of BADAN fluorescence was determined according to the procedure described previously (Kuznetsova et al., 2012b). Quinine sulfate in 0.1 M H_2_SO_4_ was used as a reference solution. The measurements were made at 23°C with micro-cells (10 × 10 × 4 mm; Starna, Great Britain).

### Determination of protein-ligand dissociation constant

The fluorescence intensity of protein solution in the ligand presence can be determined by equation

(1) \begin{eqnarray*} \displaystyle I({C}_{0})={\alpha }_{F}({C}_{0}){I}_{F}+{\alpha }_{B}({C}_{0}){I}_{B}&&\displaystyle \end{eqnarray*}

where *I_F_* and *I_B_* are the fluorescence intensity of protein in free state and bounded with ligand, respectively, and *α_F_*(*C*_0_) and *α_B_*(*C*_0_) are the relative fraction of this protein states in the solution at concentration of added ligand *C*_0_, *α_F_*(*C*_0_) + *α_B_*(*C*_0_) = 1. Thus, the fraction of bounded protein is determined as:

(2) \begin{eqnarray*} \displaystyle {\alpha }_{B}=\frac{I({C}_{0})-{I}_{F}}{{I}_{B}-{I}_{F}}=\frac{{C}_{b}}{{C}_{p}}&&\displaystyle \end{eqnarray*}

where *C_p_* is the total protein concentration and *C_b_* is the concentration of protein bounded with ligand. The dissociation constant, *K_d_* can be expressed as follows (Kuznetsova et al., 2012a):

(3) \begin{eqnarray*} \displaystyle {K}_{d}=\frac{[\text{receptor}]\times [\text{ligand}]}{[\text{complex}]}=\frac{({C}_{p}-{C}_{b})\times {C}_{f}}{{C}_{b}}&&\displaystyle \end{eqnarray*}

where *C_f_* is concentration of free ligand, which can be calculated from the equation:

(4) \begin{eqnarray*} \displaystyle {C}_{b}={C}_{0}-{C}_{f}&&\displaystyle \end{eqnarray*}

here *C*_0_ is concentration of added ligand. Eliminating *C_f_* from the Eq. (3), we can obtain the next equation for *C_b_*:

(5) \begin{eqnarray*} \displaystyle {C}_{b}=\frac{({K}_{d}+{C}_{p}+{C}_{0})-\sqrt{({K}_{d}+{C}_{p}+{C}_{0})^{2}-4{C}_{p}\times {C}_{0}}}{2}.&&\displaystyle \end{eqnarray*}

Combining the Eqs. (2) and (5), we have the equation for definition of *K_d_*, using the difference of fluorescence intensity of mutant proteins in the presence and in the absence of glucose:

(6) \begin{eqnarray*} \displaystyle I({C}_{0})={I}_{F}+({I}_{B}-{I}_{F})\times \frac{({K}_{d}+{C}_{p}+{C}_{0})-\sqrt{({K}_{d}+{C}_{p}+{C}_{0})^{2}-4{C}_{p}\times {C}_{0}}}{2{C}_{p}}.&&\displaystyle \end{eqnarray*}

The wavelength of registration was chosen as that of the maximal difference in the fluorescence intensity of the ligand-free and bound states of the studied protein. Approximation of experimental data was performed via the nonlinear regression method using Sigma Plot program.

## Results and Discussion

### The dissociation constant of the GGBP/H152C-BADAN-Glc complex

**Figure 1:**
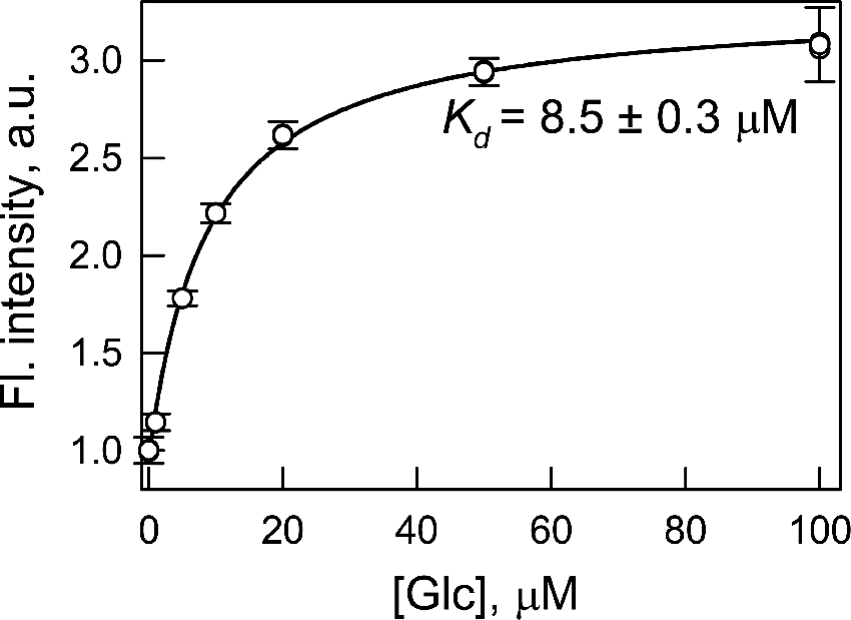
Determination of the dissociation constant (*K_d_*) of the complex of GGBP/H152C-BADAN with glucose (Glc). The excitation wavelength was 387 nm, the emission wavelength was 545 nm.

The dissociation constant of wild type GGBP with glucose is near 1 µM (Tolosa et al., 1999). We determined the dissociation constant of the GGBP/H152C complex with glucose by intrinsic protein fluorescence (without extrinsic dye) as 5.6 µM (data not shown). The replacement of histidine residue 152 with cysteine increases the dissociation constant for the complex of the protein with glucose by approximately 5 times. This effect can be attributed to the break of the hydrogen bond between residue 152 of GGBP and the sixth oxygen atom of the glucose molecule (Vyas, Vyas & Quiocho, 1988) as a result of the replacement of histidine to cysteine. The labeling of GGBP/H152C with BADAN increases the protein–glucose dissociation constant to 8.5 µM. This dissociation constant was determined by BADAN fluorescence intensity at the interaction of GGBP/H152C-BADAN with glucose (Fig. 1). The changing of dissociation constant of GGBP/H152C with glucose which follows protein labeling with BADAN may occur because dye disturbs the configuration correspondence between the protein active site and the glucose molecule. Biosensor system will continuously response to the changes in sugar content if the protein–glucose complex dissociation constant numerically corresponds to the desired range of glucose concentrations. The dissociation constant of complex GGBP/H152C-BADAN with glucose numerically is significantly less than the glucose concentration in the blood and interstitial fluids of both healthy people (3–8 mM) and patients with diabetes (Renard, 2005). A numerical accordance between the dissociation constant value of the GGBP/H152C-BADAN complex with glucose and the sugar concentration in the test medium can be achieved using transdermal methods of glucose extraction (such as reverse iontophoresis (Rhee et al., 2007), sonophoresis (Kost et al., 2000), fast microdialysis (Heinemann, 2003), or laser poration (Ge, Rao & Tolosa, 2008)). In this case, there is a dilution of the glucose concentration by three orders in the extractive fluids compared to the physiological values (Oliver et al., 2009).

### The kinetics of the formation of the GGBP/H152C-BADAN-Glc complex

**Figure 2:**
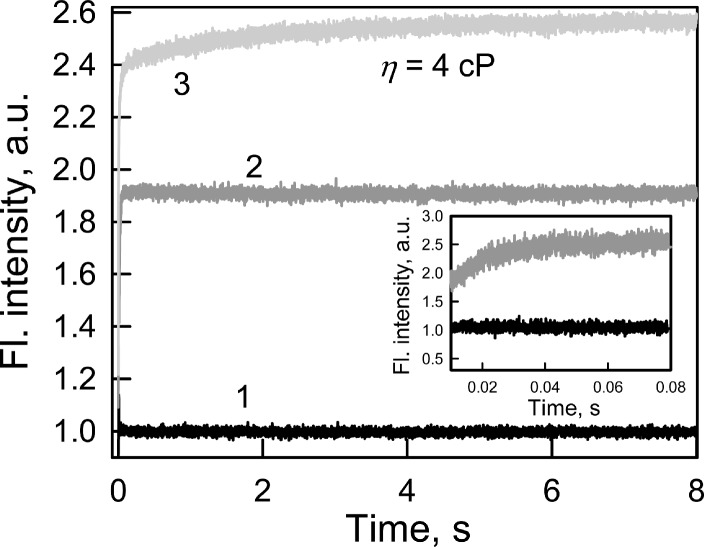
The kinetics of Glc binding to GGBP/H152C-BADAN in solutions with different viscosities. Glc concentration was 20 mM. The excitation wavelength was 405 nm. Curve 1, 2 and 3 represents the control, Glc binding to GGBP/H152C-BADAN in a PBS solution, and in PBS-glycerol solution with viscosity equal to 4.0 cP. Inset: The kinetic curves 1 and 2 in interval from 0.01 to 0.08 s are given.

Every biosensor system should respond quickly to changes in the content of the analyte in analyzed medium. In this regard, the fluorescence kinetics of GGBP/H152C-BADAN following the introduction of glucose to the test solution was investigated (Fig. 2).

The fluorescence intensity of BADAN linked to the protein open form reaches the fluorescence intensity of the dye of the complex GGBP/H152C-BADAN with glucose via 0.08 s after the addition of abundance of sugar (molar concentration of glucose tenfold higher than the molar concentration of protein) to the analyzed solution. This indicates a low value of the energy barrier between the open and closed forms of GGBP/H152C.

The viscosity of the medium can affect the rate of ligand-receptor complex formation (Bongrand, 1999). The formation of the protein–glucose complex in solution with a viscosity corresponding to that of blood (4 cP; Dintenfass, 1985) is fast enough: it takes not more than 3 s (Fig. 2) to allow for the use of the mutant form GGBP/H152C-BADAN in the determination of glucose concentrations in viscous media.

### Effect of solution on the fluorescence quantum yield of GGBP/H152C-BADAN

BADAN is a solvatochromic dye (Koehorst, Spruijt & Hemminga, 2008; Owenius et al., 1999) which fluorescent characteristics are largely determined by the polarity of the medium. We have shown that changes in the polarity of the medium have a significant effect on the fluorescence quantum yield of the free dye. Value of BADAN quantum yield is increased by 1.7 times at the transition from a polar environment in mixed solvent of acetonitrile/water at a ratio 1:100 to a less polar environment (acetonitrile).

It was shown that restriction of the mobility of the dye may also have a significant effect on the BADAN fluorescence quantum yield. When the dye links to protein its fluorescence quantum yield increases by approximately 20 times compared with that of the free dye in the mixed acetonitrile/water solvent. This increase may indicate that there exists a mechanism of non-radiative deactivation of the excited state of the dye which is associated with the mobility of propanoyl and naphthalene fragments of the BADAN molecule (Koehorst, Spruijt & Hemminga, 2008) relative to one another.

In this case, the expression for the dye quantum yield takes the following form:

(7) \begin{eqnarray*} \displaystyle q=\frac{{k}_{f}}{{k}_{f}+{k}_{\varphi }+{k}_{{\varphi }_{0}}}&&\displaystyle \end{eqnarray*}

where *k_f_* is the rate constant of the deactivation process of the excited state of the dye with radiation, *k*_*ϕ*_ is the rate constant of the non-radiative deactivation of the excited state of the fluorophore molecule due to the mobility of parts of the molecule relative to each other, *k*_*φ*_0__ is the rate constant of the non-radiative deactivation of the excited state of BADAN not conjugated with the mobility of parts of the molecule relative to each other.

It can be assumed that according to the Debye–Stokes–Einstein law, *k*_*ϕ*_ is proportional to the ratio of the temperature of the solvent to its viscosity (Loutfy & Arnold, 1982):

(8) \begin{eqnarray*} \displaystyle {k}_{\phi }\sim \frac{T}{\eta }.&&\displaystyle \end{eqnarray*}

Then Eq. (7) can be written as follows:

(9) \begin{eqnarray*} \frac{1}{q}=1+a+b\frac{T}{\eta }, \end{eqnarray*}

where $a=\frac{{k}_{{\varphi }_{0}}}{{k}_{f}}=(1/q-1)$ at *T* → 0 and *η* → ∞, *b* is the proportionality coefficient.

**Figure 3:**
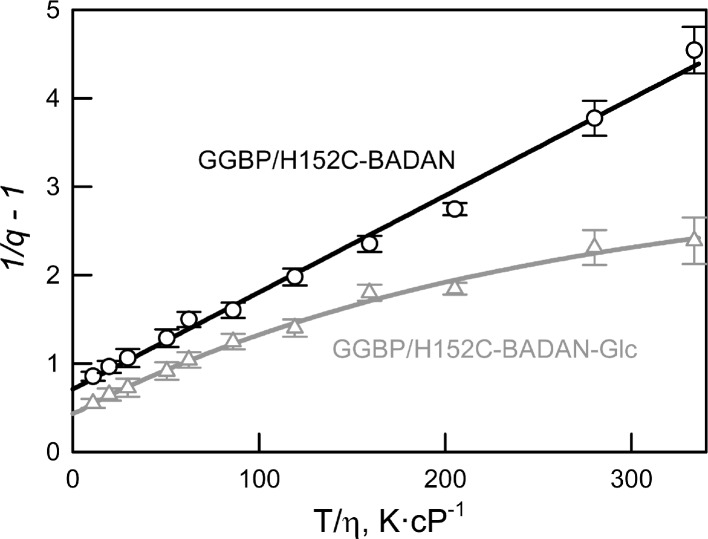
The effect of solvent viscosity and temperature on the fluorescence quantum yield of the GGBP/H152C-BADAN in the open (circles) and closed (triangles) forms. The excitation wavelength was 387 nm.

We have shown that the dependence of the fluorescence quantum yield of BADAN linked to the open form of GGBP/H152C on viscosity and temperature (expressed in coordinates 1/*q*−1 on the *T*/*η* ratio) is linear (Fig. 3). This finding confirms the existence of a mechanism of non-radiative deactivation of the excited state of the dye connected with the mobility of the propanoyl and naphthalene fragments of BADAN molecule relative to each other. The dependence of 1/*q*−1 of the BADAN fluorescence in the complex GGBP/H152C-BADAN-Glc on the *T*/*η* ratio is a nonlinear curve (Fig. 3). Probably, it is explained by the reduced accessibility of BADAN to the solvent molecules in the complex of GGBP/H152C with the ligand.

### GdnHCl and urea-induced conformational changes of GGBP/H152C-BADAN in open and closed forms

**Figure 4:**
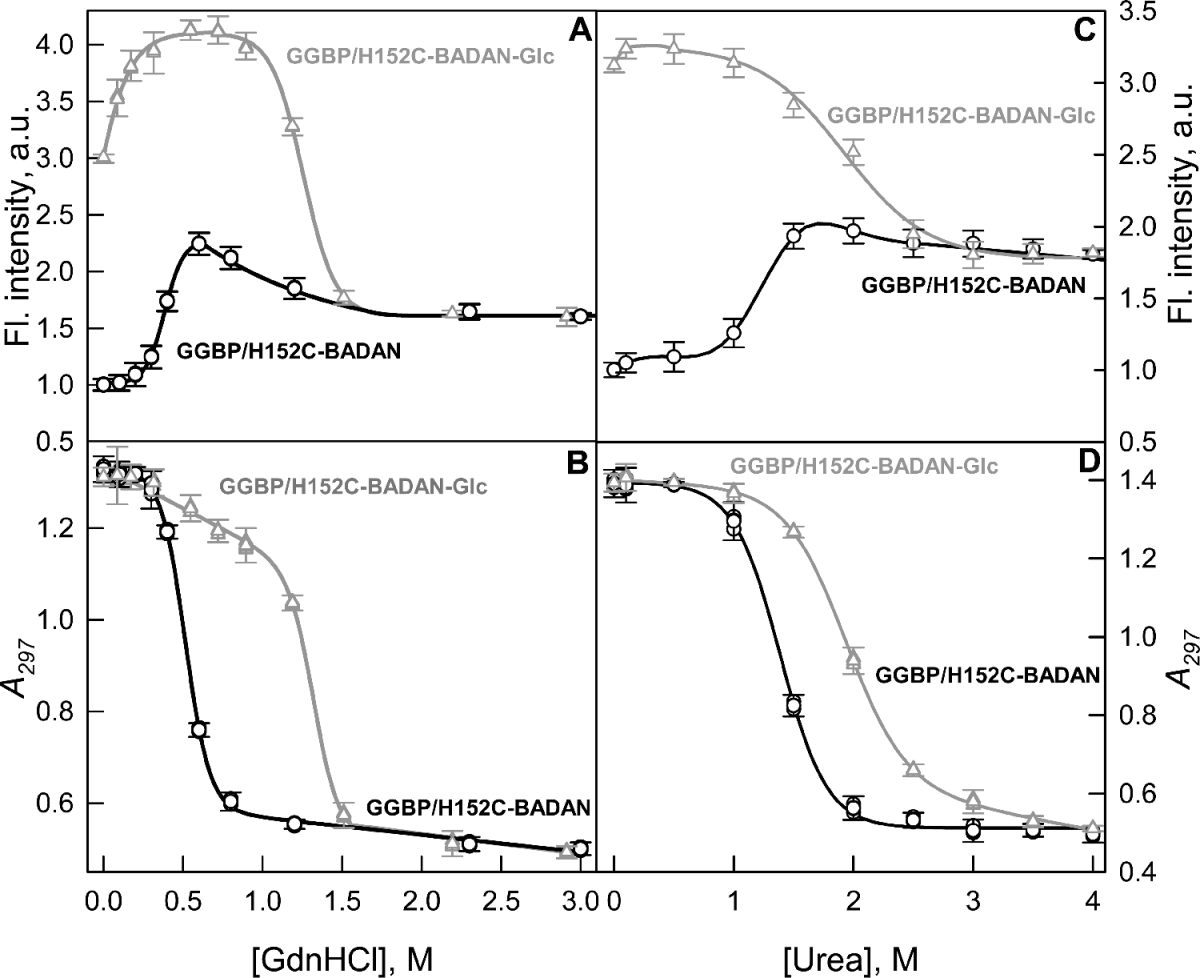
GdnHCl-induced (A, B) and urea-induced (C, D) conformational changing of GGBP/H152C-BADAN structure in open (circles) and closed forms (triangles). (A) and (C) represents the dependence of the fluorescence intensity (the excitation wavelength was 387 nm, the emission wavelength was 545 nm) on the denaturants concentration. (B) and (D) represents the dependence of the parameter *A* = *I*_320_/*I*_365_ (*I*_320_ and *I*_365_ fluorescence intensities recorded at 320 and 365 nm, respectively) on the denaturants concentration (the excitation wavelength was 297 nm).

The stability of the mutant form GGBP/H152C was evaluated by studying the conformational changes of GGBP/H152C-BADAN in open and closed forms induced by chemical denaturants: GdnHCl and urea (Fig. 4). The analysis of intrinsic UV fluorescence (Figs. 4B and 4D) show that the structure of open form of GGBP/H152C-BADAN changes at relatively low concentrations of denaturants (the middle of the transition between the GGBP/H152C-BADAN native and unfolded state is near 0.5 M GdnHCl and 1.25 M urea, respectively). This finding indicates a relatively low stability of target protein. Complex formation with glucose leads to a significant stabilization of the GGBP/H152C structure. The middle of the transition between the native and unfolded state of GGBP/H152C-BADAN-Glc is shifted to higher concentrations of GdnHCl and urea (the mid point of transition corresponds to 1.4 M of GdnHCl and 2.0 M of urea, respectively).

The dependences of the fluorescence intensity of BADAN in GGBP/H152C-BADAN and its complex with glucose on the denaturants concentration provide information about the local changes in protein structure in the vicinity of GGBP/H152C glucose binding center.

Interestingly, the dependences of the fluorescence intensity of BADAN on denaturant concentration differ significantly from that of intrinsic protein fluorescence. The dependences of intrinsic protein fluorescence on the denaturant concentrations are similar for open and closed forms and both indicate a one-step protein unfolding.

At the same time the dependencies of fluorescence intensity of BADAN for GGBP/H152C-BADAN and GGBP/H152C-BADAN-Glc on the concentration of denaturants differ significantly (Figs. 4A and 4C). Furthermore, the dependencies of fluorescence intensity of BADAN in GGBP/H152C-BADAN form indicates the existence of two processes. Firstly, the fluorescence intensity increases with the increase of denaturant concentration from 0.3 to 0.6 M of GdnCHl and from 1.0 to 1.7 M of urea and only then fluorescence intensity decreases. The maxima of these curves nearly correspond to the mid points of the dependences of *A* parameter of intrinsic fluorescence on denaturant concentration. So, it means that protein denaturation consists of two processes: first, conformational changes lead to more nonpolar environment in the vicinity of BADAN and, second, protein unfolds. The initial stage of denaturation may be due to the different stabilities of N and C-domains of GGBP. Previously, by differential scanning calorimetry it was shown that the domains of the protein unfold at different temperatures during the heat-induced denaturation of GGBP (Stepanenko et al., 2011a). Successive unfolding of N and C-terminal domains of GGBP/H152C may promote sophisticated changes of the dye environment. These processes can be accompanied by some convergence of N and C-terminal domains of GGBP/H152C. Analysis of GGBP (glucose-free form) structure shows that this protein in solution exists in dynamic equilibrium between the open and semi-closed forms (Borrok, Kiessling & Forest, 2007). Perhaps during protein denaturation dynamic equilibrium shifts towards the formation of a semi-closed form GGBP. Since BADAN is localized in the slit between the domains of GGBP/H152C, the microenvironment of the dye can become less polar and more densely packed. As would be expected, these processes do not affect the fluorescence of BADAN in GGBP/H152C-BADAN-Glc, when its microenvironment is already packed. Except the range of small GdnHCl concentrations (0–0.3 M GdnHCl), fluorescence of BADAN in GGBP/H152C-BADAN-Glc does not change with the increase of denaturant concentrations until protein unfolding.

**Figure 5:**
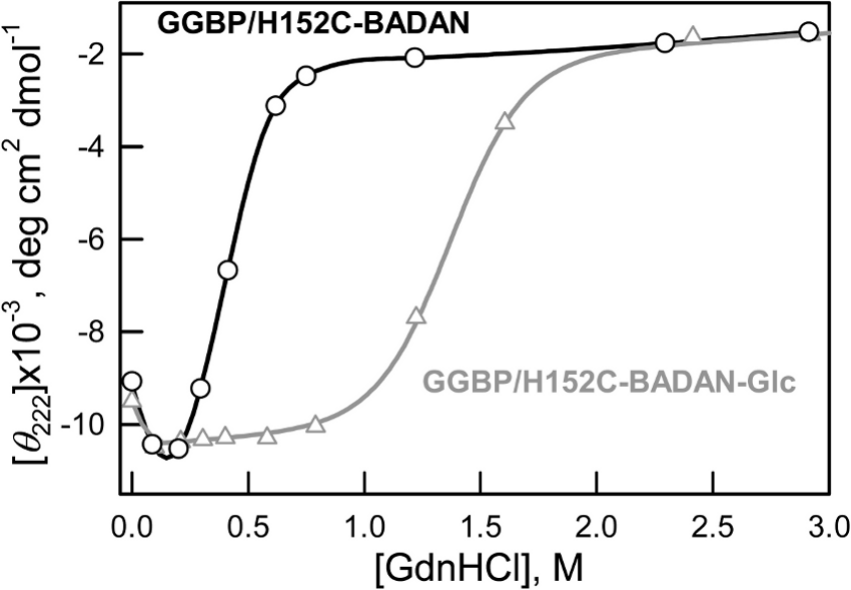
Changes of ellipticity at 222 nm of GGBP/H152C-BADAN and GGBP/H152C-BADAN–Glc on the GdnHCl concentration.

Earlier it was shown that low concentrations of GdnHCl have a stabilizing effect on the number of proteins in which interactions with Gdn+ ion remove local stresses in their structure (Kuznetsova et al., 2002; Povarova, Kuznetsova & Turoverov, 2010; Stepanenko et al., 2004; Verkhusha et al., 2003). In our case, the increase of CD signal in the range of 0–0.3 M GdnHCl of both open and closed forms of GGBP/H152C indicate their compact structure in these conditions (Fig. 5).

It’s interesting that this local compaction of protein structure affects BADAN in GGBP/H152CBADAN-Glc form but not in GGBP/H152C-BADAN. Perhaps BADAN linked to the open form of GGBP/H152C is rather available to the solvent, so that the overall compaction of protein structure does not affect its microenvironment.

Our assumptions concerning the affects of small concentrations of GdnHCl are confirmed by the absence of fluorescence intensity growth of GGBP/H152C-BADAN-Glc at low concentrations of urea (Fig. 4C) and invariance fluorescence characteristics of free dye in solution with GdnHCl in low concentration (data not shown).

### The dependence of the fluorescence characteristics of GGBP/H152C-BADAN in its open and closed forms on the pH and ionic strength of the solution

Changing pH and ionic strength are two factors that may have a significant effect on the fluorescence characteristics of GGBP/H152C-BADAN used for monitoring glucose in biological fluids. As a rule, the pH in biological fluids is in neutral range, the primary source of ions in such fluids is NaCl which ‘physiological’ concentration is about 0.15 M.

**Figure 6:**
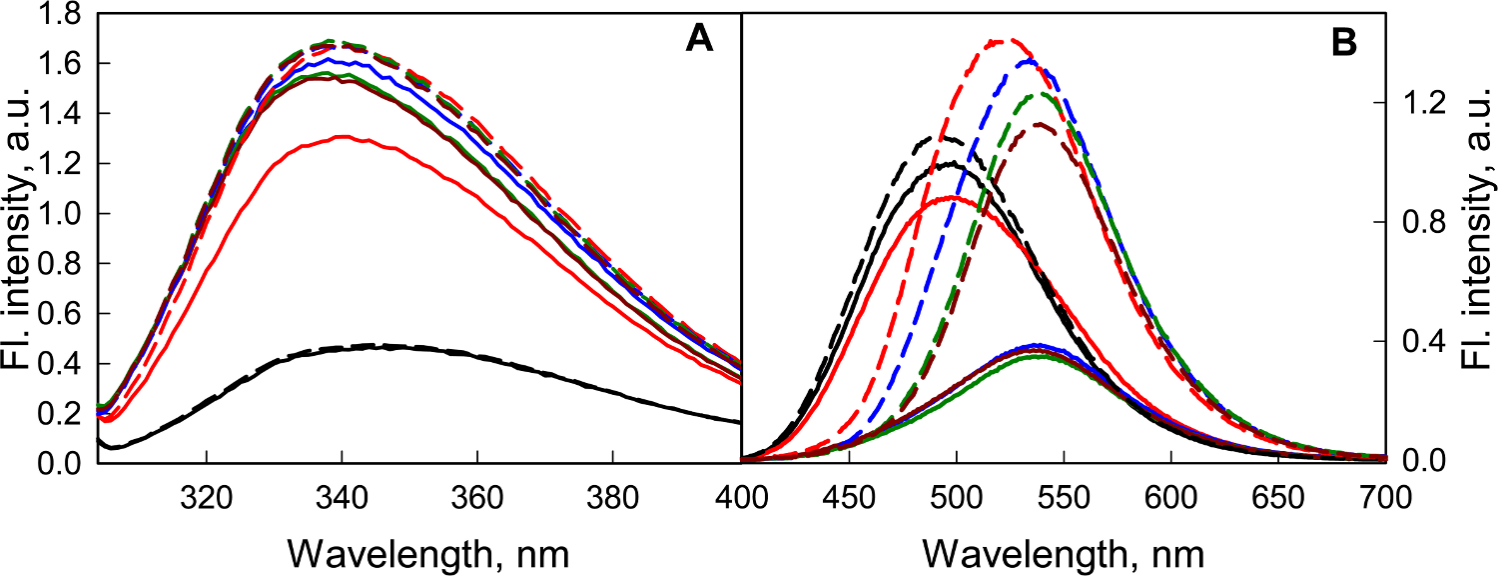
The effect of the pH solution on the fluorescent characteristics of GGBP/H152C-BADAN in the open (the solid curves) and closed (the dashed curves) forms. (A) The excitation wavelength was 297 nm. (B) The excitation wavelength was 387 nm. The pH values of the solution were 2.8 (black curves), 4.2 (red curves), 6.4 (blue curves), 7.4 (dark green curves) and 9.6 (dark red curves).

It was shown that a weak acidification of the solution has no significant effect on either the structure of the GGBP/H152C or the fluorescent characteristics of the dye linked to it (Fig. 6). According to the BADAN fluorescence (increase of fluorescence intensity of the dye at the complex formation of GGBP/H152C with glucose), the ability of GGBP/H152C to bind glucose was maintained until about pH 4.2 (Fig. 6B). Upon further acidification of the medium, the interaction of the protein with glucose does not lead to significant changes in the fluorescence intensity and fluorescence spectra of GGBP/H152C-BADAN. This finding indicates that under these conditions, GGBP/H152C loses the ability to bind glucose. Decreasing the pH of solution from 4.2 to 2.8 results in a loss of the native structure of GGBP/H152C. This is proved by a decrease in the intensity of the intrinsic UV fluorescence of GGBP/H152C (Fig. 6A). At that it was observed an increase in the fluorescence intensity of the BADAN and a blue-shift of the BADAN fluorescence spectrum compared that in solutions with a neutral pH (Fig. 6B).

**Figure 7:**
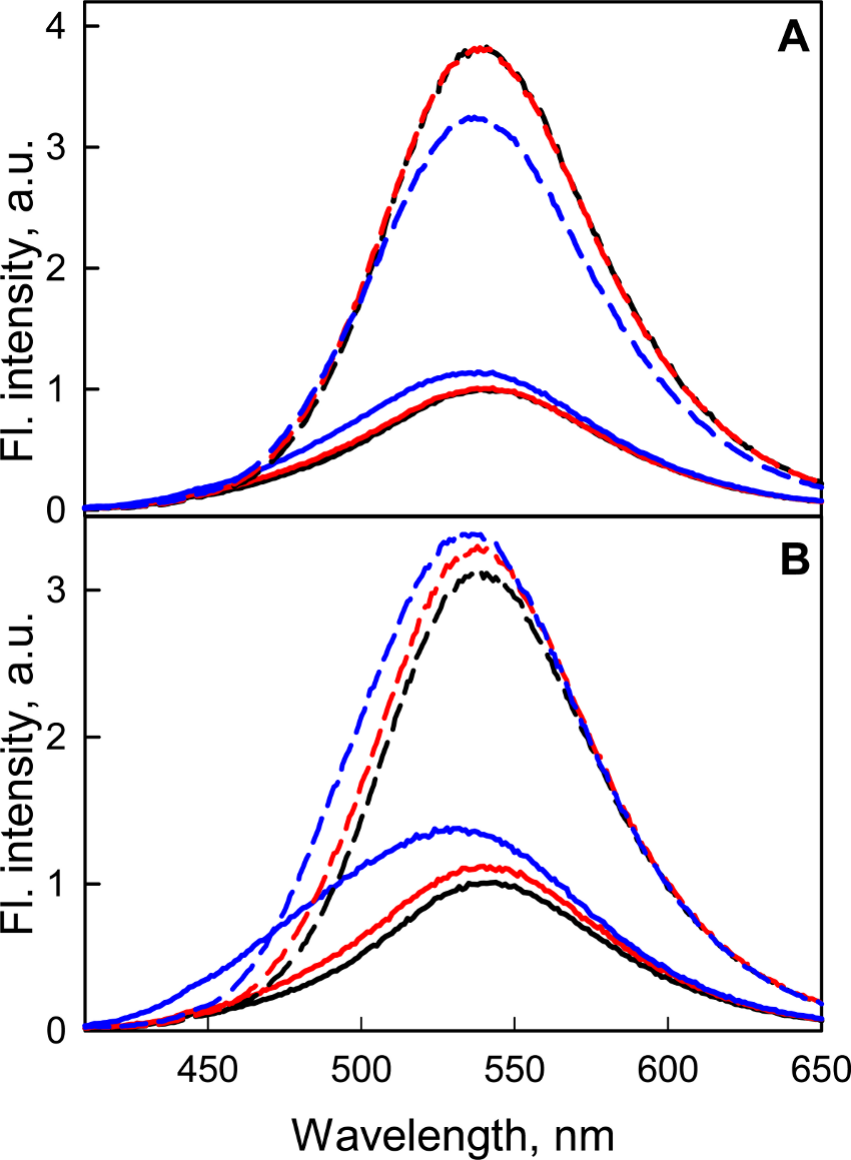
The effect of NaCl and MgCl_2_ on the fluorescent characteristics of GGBP/H152C-BADAN in open (solid curves) and closed (dashed curves) forms. (A) Fluorescence spectra of GGBP/H152C-BADAN in solutions with 0.8 M NaCl (red curve) and 2.5 M NaCl (blue curve). (B) Fluorescence spectra of GGBP/H152C-BADAN in solutions with 0.1 M MgCl_2_ (red curve) and 0.5 M MgCl_2_ (blue curve). The fluorescence spectra of GGBP/H152C-BADAN in PBS solution appear in black. (The excitation wavelength was 387 nm.)

At the transition from neutral to alkaline pH ranges, the intrinsic UV fluorescence of GGBP/H152C and the fluorescence of the dye linked to the protein changed slightly (Fig. 7), but the ability of GGBP/H152C-BADAN to bind glucose is retained. An analysis of the effect of changing pH on fluorescence characteristics of the GGBP/H152C-BADAN in its open and closed forms indicates the invariability of fluorescence characters of GGBP/H152C in the limits of pH physiological values. The transition from neutral to acidic pH regions contributes to the loss of the native structure of the protein. To examine the effect of ionic strength of solution and presence of divalent metal ions on the fluorescence characteristics of BADAN linked to GGBP/H152C we studied the dependence of its fluorescence intensity on the concentrations of NaCl and MgCl_2_ in solution. Changing the ionic strength of the solution has no essential effect on the fluorescence characteristics of the protein and the dye linked to GGBP/H152C (Fig. 7). Only slight decrease in the intensity of protein intrinsic fluorescence and BADAN fluorescence intensity in comparison with these characteristic of protein in PBS was observed for of GGBP/H152C in solutions containing about 2.5 M NaCl and 0.5 M MgCl_2_. Therefore, GGBP/H152C does not lose the ability to bind glucose in physiologic range of these ions’ content.

## Conclusion

The obtained data allows us to conclude that the GGBP/H152C-BADAN mutant form could have limited use as a sensitive element for a glucose biosensor system for the determination of sugar concentrations in biological fluids extracted using transdermal technologies, but cannot be used for direct determination of the glucose concentration in biological fluids. In principle, using point mutagenesis one can try to solve this problem.

The desired form should have both three orders of magnitude greater dissociation constant with glucose, but should also preserve a significant difference in the fluorescence intensity of the dye bound to open and closed forms of protein. The mutant form GGBP/H152C/A213R/L238S is one such promising structure (Khan, Saxl & Pickup, 2010).

Experiments on protein denaturation by chemical denaturants such as GdnHCl and urea indicate that GGBP has a relatively low stability. For prolonged continuous glucose monitoring the use of more stable sensitive element is advisable. Sugar-binding proteins from thermophilic bacteria are good candidates for this purpose.

## References

[ref-1] Amiss TJ, Sherman DB, Nycz CM, Andaluz SA, Pitner JB (2007). Engineering and rapid selection of a low-affinity glucose/galactose-binding protein for a glucose biosensor. Protein Science.

[ref-2] Bongrand P (1999). Ligand–receptor interactions. Reports on Progress in Physics.

[ref-3] Borrok MJ, Kiessling LL, Forest KT (2007). Conformational changes of glucose/galactose-binding protein illuminated by open, unliganded, and ultra-high-resolution ligand-bound structures. Protein Science.

[ref-4] Dintenfass L (1985). Blood viscosity, hyperviscosity, and hyperviscosaemia.

[ref-5] Ge X, Rao G, Tolosa L (2008). On the possibility of real-time monitoring of glucose in cell culture by microdialysis using a fluorescent glucose binding protein sensor. Biotechnology Progress.

[ref-6] Ge X, Tolosa L, Rao G (2004). Dual-labeled glucose binding protein for ratiometric measurements of glucose. Analytical Chemistry.

[ref-7] Heinemann L (2003). Continuous glucose monitoring by means of the microdialysis technique: underlying fundamental aspects. Diabetes Technology & Therapeutics.

[ref-8] Katsube T, Katoh M, Maekawa H, Hara M, Yamaguchi S, Uchida N, Shimomura T (1990). Stabilization of an FET glucose sensor with the thermophilic enzyme glucokinase. Sensors and Actuators B: Chemical.

[ref-9] Khan F, Gnudi L, Pickup JC (2008). Fluorescence-based sensing of glucose using engineered glucose/galactose-binding protein: a comparison of fluorescence resonance energy transfer and environmentally sensitive dye labelling strategies. Biochemical and Biophysical Research Communications.

[ref-10] Khan F, Saxl TE, Pickup JC (2010). Fluorescence intensity- and lifetime-based glucose sensing using an engineered high-Kd mutant of glucose/galactose-binding protein. Analytical Biochemistry.

[ref-11] Koehorst RB, Spruijt RB, Hemminga MA (2008). Site-directed fluorescence labeling of a membrane protein with BADAN: probing protein topology and local environment. Biophysical Journal.

[ref-12] Kost J, Mitragotri S, Gabbay RA, Pishko M, Langer R (2000). Transdermal monitoring of glucose and other analytes using ultrasound. Nature Medicine.

[ref-13] Kuznetsova IM, Stepanenko Olga V, Turoverov KK, Zhu L, Zhou JM, Fink AL, Uversky VN (2002). Unraveling multistate unfolding of rabbit muscle creatine kinase. Biochimica et Biophysica Acta/Protein Structure and Molecular Enzimology.

[ref-14] Kuznetsova IM, Sulatskaya AI, Povarova OI, Turoverov KK (2012a). Reevaluation of ANS binding to human and bovine serum albumins: key role of equilibrium microdialysis in ligand-receptor binding characterization. PLoS ONE.

[ref-15] Kuznetsova IM, Sulatskaya AI, Uversky VN, Turoverov KK (2012b). A new trend in the experimental methodology for the analysis of the thioflavin T binding to amyloid fibrils. Molecular Neurobiology.

[ref-16] Kuznetsova IM, Yakusheva TA, Turoverov KK (1999). Contribution of separate tryptophan residues to intrinsic fluorescence of actin. Analysis of 3D structure. FEBS Letters.

[ref-17] Loutfy RO, Arnold BA (1982). Effect of viscosity and temperature on torsional relaxation of molecular rotors. The Journal of Physical Chemistry.

[ref-18] Oliver NS, Toumazou C, Cass AE, Johnston DG (2009). Glucose sensors: a review of current and emerging technology. Diabetic Medicine.

[ref-19] Owenius R, Osterlund M, Lindgren M, Svensson M, Olsen OH, Persson E, Freskgard PO, Carlsson U (1999). Properties of spin and fluorescent labels at a receptor–ligand interface. Biophysical Journal.

[ref-20] Povarova OI, Kuznetsova IM, Turoverov KK (2010). Differences in the pathways of proteins unfolding induced by urea and guanidine hydrochloride: molten globule state and aggregates. PLoS ONE.

[ref-21] Renard E (2005). Monitoring glycemic control: the importance of self-monitoring of blood glucose. American Journal of Medicine.

[ref-22] Rhee SY, Chon S, Koh G, Paeng JR, Oh S, Woo JT, Kim SW, Kim JW, Kim YS (2007). Clinical experience of an iontophoresis based glucose measuring system. Journal of Korean Medical Science.

[ref-23] Sakaguchi-Mikami A, Taneoka A, Yamoto R, Ferri S, Sode K (2008). Engineering of ligand specificity of periplasmic binding protein for glucose sensing. Biotechnology Letters.

[ref-24] Saxl T, Khan F, Ferla M, Birch D, Pickup J (2011). A fluorescence lifetime-based fibre-optic glucose sensor using glucose/galactose-binding protein. Analyst.

[ref-25] Saxl T, Khan F, Matthews DR, Zhi ZL, Rolinski O, Ameer-Beg S, Pickup J (2009). Fluorescence lifetime spectroscopy and imaging of nano-engineered glucose sensor microcapsules based on glucose/galactose-binding protein. Biosensors and Bioelectronics.

[ref-26] Stepanenko Olga V, Fonin AV, Morozova KS, Verkhusha VV, Kuznetsova IM, Turoverov KK, Staiano M, D’Auria S (2011a). New insight in protein-ligand interactions. 2. Stability and properties of two mutant forms of the D-galactose/D-glucose-binding protein from *E. coli*. The Journal of Physical Chemistry B.

[ref-27] Stepanenko Olga V, Povarova OI, Fonin AV, Kuznetsova IM, Turoverov KK, Staiano M, Varriale A, D’Auria S (2011b). New insight into protein-ligand interactions. The case of the D-galactose/D-glucose-binding protein from Escherichia coli. The Journal of Physical Chemistry B.

[ref-28] Stepanenko Olesya V, Verkhusha VV, Kazakov VI, Shavlovsky MM, Kuznetsova IM, Uversky VN, Turoverov KK (2004). Comparative studies on the structure and stability of fluorescent proteins EGFP, zFP506, mRFP1, “dimer2”, and DsRed1. Biochemistry.

[ref-29] Tang H, Chen J, Yao S, Nie L, Deng G, Kuang Y (2004). Amperometric glucose biosensor based on adsorption of glucose oxidase at platinum nanoparticle-modified carbon nanotube electrode. Analytical Biochemistry.

[ref-30] Tolosa L, Gryczynski I, Eichhorn LR, Dattelbaum JD, Castellano FN, Rao G, Lakowicz JR (1999). Glucose sensor for low-cost lifetime-based sensing using a genetically engineered protein. Analytical Biochemistry.

[ref-31] Turoverov KK, Biktashev AG, Dorofeiuk AV, Kuznetsova IM (1998). A complex of apparatus and programs for the measurement of spectral, polarization and kinetic characteristics of fluorescence in solution. Tsitologiia.

[ref-32] Turoverov KK, Kuznetsova IM (2003). Intrinsic fluorescence of actin. Journal of Fluorescence.

[ref-33] Verkhusha VV, Kuznetsova IM, Stepanenko Olesya V, Zaraisky AG, Shavlovsky MM, Turoverov KK, Uversky VN (2003). High stability of Discosoma DsRed as compared to Aequorea EGFP. Biochemistry.

[ref-34] Vyas NK, Vyas MN, Quiocho FA (1988). Sugar and signal-transducer binding sites of the *Escherichia coli* galactose chemoreceptor protein. Science.

[ref-35] Ye K, Schultz JS (2003). Genetic engineering of an allosterically based glucose indicator protein for continuous glucose monitoring by fluorescence resonance energy transfer. Analytical Chemistry.

[ref-36] Yesylevskyy SO, Kharkyanen VN, Demchenko AP (2006). The change of protein intradomain mobility on ligand binding: is it a commonly observed phenomenon?. Biophysical Journal.

